# Supplementary material for: Jade1 and the HBO1 histone acetyltransferase complex are spatial-selective cofactors of the pluripotency transcription factor Oct4
Source: J Biol Chem. 2025 Oct 24;301(12):110859. doi: 10.1016/j.jbc.2025.110859 (PMC12664036; doi:10.1016/j.jbc.2025.110859)
Supplement: Supporting Figures [file mmc6.docx]

Uncropped immunoblot images for

**Jade1 and the HBO1 complex are spatial-selective cofactors of Oct4**

Yifan Wu,^1,2,¶^ Asit K. Manna,^1,2,¶^ Li Li,^1,2^, Zuolian Shen^1,2^, Hiroshi Handa,^2,3,4^ Mahesh B. Chandrasekharan,^2,5^ and Dean Tantin^1,3,*^

^1^Department of Pathology, University of Utah, School of Medicine, Salt Lake City, UT 84112, USA

^2^Huntsman Cancer Institute, University of Utah, School of Medicine, Salt Lake City, UT 84112, USA

^3^Department of Oncological Sciences, University of Utah, Salt Lake City, UT 84112, USA

^4^Center for Future Medical Research, Institute of Medical Science, Tokyo Medical University, Shinjuku, Shinjuku-Ku, Tokyo, 160-8402, Japan

^5^Department of Radiation Oncology, University of Utah, School of Medicine, Salt Lake City, UT 84112, USA

*Correspondence: [dean.tantin@path.utah.edu](mailto:dean.tantin@path.utah.edu).

**^¶^**Co-first authors
